# 3D-BoxSup: Positive-Unlabeled Learning of Brain Tumor Segmentation Networks From 3D Bounding Boxes

**DOI:** 10.3389/fnins.2020.00350

**Published:** 2020-04-28

**Authors:** Yanwu Xu, Mingming Gong, Junxiang Chen, Ziye Chen, Kayhan Batmanghelich

**Affiliations:** ^1^Department of Biomedical Informatics, University of Pittsburgh, Pittsburgh, PA, United States; ^2^School of Mathematics and Statistics, The University of Melbourne, Melbourne, VIC, Australia; ^3^School of Compuer Science, Wuhan University, Wuhan, China

**Keywords:** brain tumor segmentation, deep learning, weakly-supervised, 3D bounding box, positive-unlabeled learning

## Abstract

Accurate segmentation is an essential task when working with medical images. Recently, deep convolutional neural networks achieved a state-of-the-art performance for many segmentation benchmarks. Regardless of the network architecture, the deep learning-based segmentation methods view the segmentation problem as a supervised task that requires a relatively large number of annotated images. Acquiring a large number of annotated medical images is time consuming, and high-quality segmented images (i.e., strong labels) crafted by human experts are expensive. In this paper, we have proposed a method that achieves competitive accuracy from a “weakly annotated” image where the weak annotation is obtained via a 3D bounding box denoting an object of interest. Our method, called “3D-BoxSup,” employs a positive-unlabeled learning framework to learn segmentation masks from 3D bounding boxes. Specially, we consider the pixels outside of the bounding box as positively labeled data and the pixels inside the bounding box as unlabeled data. Our method can suppress the negative effects of pixels residing between the true segmentation mask and the 3D bounding box and produce accurate segmentation masks. We applied our method to segment a brain tumor. The experimental results on the BraTS 2017 dataset (Menze et al., 2015; Bakas et al., 2017a,b,c) have demonstrated the effectiveness of our method.

## 1. Introduction

Gliomas are one of the most common brain tumors in adults. They can be categorized into different levels of aggressiveness, including High-Grade Gliomas (HGG) and Lower Grade Gliomas (LGG) (Louis et al., 2016). Gliomas consist of heterogeneous histological sub-regions, including peritumoral edema, the necrotic core, as well as the enhancing and non-enhancing tumor core (Menze et al., 2015). Magnetic Resonance Imaging (MRI) of brain tumors is commonly used to evaluate tumor progression and plan treatments. An MRI usually contains multi-modal data, such as T1-weighted, T2-weighted, contrast enhanced T1-weighted (T1ce), and Fluid Attenuation Inversion Recovery (FLAIR) images, which provide complementary information for analysis of brain tumors.

The automatic segmentation of brain tumors and subregions is a crucial pre-treatment step for the characterization and sub-typing ofgliomas. This is a challenging problem because tumors vary in shape and size across patients and may have low contrast in some modalities. Recently, deep convolutional neural network (CNN)-based methods have achieved new records in brain tumor segmentation. Most of these methods are extensions of the U-Net structure (Ronneberger et al., 2015; Çiçek et al., 2016) in various ways (Isensee et al., 2017, 2018; Kamnitsas et al., 2017a,b; Wang et al., 2017; Li et al., 2018). For example, some works focus on the design of new convolutional network structures, such as using a mix between convolutional kernels and modifying the down-sampling strategy (Havaei et al., 2015; Kamnitsas et al., 2017b). Other works have aimed to improve the method of fusing multi-modal information. For example, Wang et al. (2017) suggested a patch-based framework combined with multi-view fusion techniques to reduce false positive segmentation. Kamnitsas et al. (2017a) proposed another fusion method through aggregation of predictions from a wide range of methods. The overall approach is more robust and reduces the risk of over-fitting to a particular dataset.

A key problem of CNN-based segmentation methods is the requirement of accurate pixel/voxel-level annotations. However, annotating a 3D image at the voxel level requires human expertise and is expensive and time consuming. Motivated by a recent work in weakly supervised segmentation in natural 2D images (Dai et al., 2015), we proposed to learn the segmentation network from 3D bounding box annotations. As pointed out in Dai et al. (2015), boxing out the object location is about 15 times faster than drawing the segmented mask (Dai et al., 2015). In 3D MRI images, the burden of annotating voxels is much higher than that of annotating 2D images because the number of voxels increases exponentially with image dimension. However, the cost of 3D bounding box annotations is comparable to that of 2D bounding boxes. Therefore, learning from 3D bounding boxes is valuable for brain tumor segmentation.

In this paper, we have investigated how to train a segmentation network from coarse but easily accessible 3D bounding box annotation. The main difficulty comes from the inaccurate annotations inside the bounding box. More specifically, the region bounded by a 3D bounding box contains tumor voxels as well background voxels. If one simply considers the voxels inside and outside of the bounding box as two classes, i.e., tumor and non-tumor; the non-tumor voxels inside the bounding box will have the wrong labels, and the learned network tends to classify the voxels outside but close to the tumor boundaries as tumor voxels. To solve this problem, we considered segmentation from 3D bounding boxes as a positive-unlabeled (PU) learning problem (Denis, 1998; Elkan and Noto, 2008) in which we consider the voxels outside of the bounding boxes as a positive class and the voxels inside the bounding box as unlabeled data. We have proposed the “3D-BoxSup” method to train a deep convolutional neural network reliably from 3D bounding box annotations with a non-negative risk estimator that is robust against overfitting (Kiryo et al., 2017). We conducted experiments on the BraTS 2017 dataset, and the results show that our method can obtain competitive accuracy by just learning from coarse bounding box annotations.

## 2. Methods

Our 3D-BoxSup method is inspired by the BoxSup method (Dai et al., 2015), which aims to segment objects from 2D bounding box annotations. BoxSup is a straightforward method to train deep CNNs from coarse box annotations. It provides a biased objective function and utilizes the updated network in turn to improve the estimated segmentation masks used for training, which means the estimated segmentation masks in the previous training epoch are used as the ground truth mask for the next epoch. However, this iterative method is not practical for the 3D patch-based method because re-calculating the segmentation mask for each volume for each epoch has a high time-cost. In addition, it is unclear whether the iterative method will finally converge to the optimal solution. To achieve a considerable performance without iteratively updating the segmentation masks, we have cast the segmentation problem as a PU learning problem and applied a non-negative PU risk estimator (Kiryo et al., 2017) as the train objective to learn the segmentation network, where we viewed the 3D box annotated region as the unlabeled data and the area outside the box as positive data. In the following section, we have outlined the base model with a biased box-learning estimator as our baseline and the unbiased-box learning method as our proposed method.

In this section, we have first introduced the basic problem setup with precise mathematical definitions. Second, we have introduced our baseline convolutional neural network architecture, used for predicting the segmentation masks. Third, we have presented how the network is usually trained if the ground truth segmentation mask is available. Last, we have described our PU learning-based 3D-BoxSup method and the corresponding algorithm.

### 2.1. Problem Setup

In the real application, the accurate segmentation mask is difficult to acquire. Thus, in this paper, we only considered the cases where we only had access to box-labeled segmentation data.

**Figure 1:**
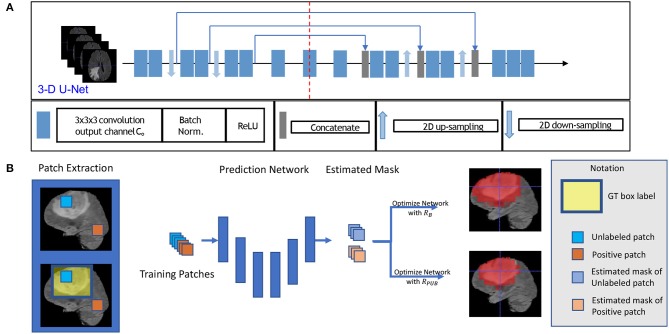
This figure shows the training model with box labeled data. The network structure is shown in sub-figure **(A)**, which is a typical 3D U-Net. The general training process is shown in sub-figure **(B)**. To be intuitive, we applied a 2D slice as our example in **(B)** in the training process, and we fed 3D patches with a 3D bounding box label to optimize our model.

Let *S*_1_ denote the annotated 3D box region where gliomas reside and *S*_0_ denote the background area outside of the bounding box. Assuming we extract 3D patches from *S*_1_ and *S*_0_ for training, which is shown in Figure 1, the label of each voxel in *S*_1_ and *S*_0_ is assigned to 1 and 0, respectively. The proportion of non-tumor voxels is denoted as $\pi \text{p}=\frac{n_{0}}{n_{0}+n_{1}}$, where *n*_1_ is the number of voxels inside the box and *n*_0_ is the number of voxels outside of the box.

### 2.2. Training Data Generation

First, for each volume of a patient, we generated a 3D bounding box to roughly cover the whole tumor (WT) area (*S*_1_), and the uncovered region was considered to be the background area *S*_0_. We show an example of the box-labeled data in Figure 1B. For convenience, we only showed the box label with a 2D yellow rectangle. It should be noted that the training label in our case is actually a 3D bounding box for each volume. In our experiment, we generated this 3D bounding box from the accurate ground truth segmentation mask, and we assumed that we did not have access to this accurate segmentation data during the training time, which was available in our testing.

We then followed the standard preprocessing step to process the original 3D input images (Bakas et al., 2018). To reduce the sensitivity to absolute pixel intensities variations, an intensity normalization step is applied to each volume of all subjects by subtracting the mean and dividing by the standard deviation so that each MR volume will have a zero mean and unit variance, which is operated to each volume dependently. In practice, as only the central region that contains the brain is used, the mean and standard deviation are estimated using this brain area; where we exclude the black area outside the brain with voxel value 0. Finally, we extract 200 patches per patient with patch size 48 × 48 × 48 in *S*_1_ and *S*_0_. If the extracted patch of *S*_1_ is partial beyond the boxed area, we pad 0 value to the exceeded part for the segmentation mask. In our experiment, we randomly selected 3D patches from area *S*_1_ and *S*_0_ with a proportion of 0.8 and 0.2, respectively. In all of the following settings, we allocated patches from *S*_1_ with the label 1 and patches from *S*_0_ with the label 0.

### 2.3. Network Structure

To build a deep network for 3D patch segmentation, we applied the 3D U-Net (Çiçek et al., 2016), consisting of an encoder and a decoder network with skip connections similar to our base model. In contrast to (Çiçek et al., 2016), we removed the last down-sampling layer and the first up-sampling layer for the LHS and RHS of the 3D U-Net, respectively. This is because the down-sampling structure would eliminate edge features of brain tumor. Our modified 3D U-Net is shown in Figure 1A.

### 2.4. Learning With Ground Truth Mask

In the fully supervised brain tumor segmentation task, accurately annotated masks were provided for training. Assuming the mask prediction function modeled by a CNN is $\hat{\text{y}}=f\left(\text{x};\theta \right)\in \text{x}\in {\mathbb{R}}^{d\times d\times d}$, where **x** ∈ ℝ^*d* × *d* × *d*^ is a randomly chosen 3D patch *U* from a patient *V*, and θ is the global trainable parameter. The ground truth patch tumor mask is **y** ∈ ℝ^*d* × *d* × *d*^. To learn the network parameters, we can apply the sigmoid function to generate probability values and cross-entropy loss function to evaluate voxel-wise prediction error. The objective function for a single value in the predicted mask can be written:

(1) $$\begin{array}{r} L_{mask}\left(y,ŷ\right)=\left(1-y\right)\cdot \log\left(\frac{1}{1+e^{-ŷ}}\right)+y\cdot \log\left(\frac{1}{1+e^{ŷ}}\right), \end{array}$$

where *y* is a single value in the ground truth mask, and $\hat{y}$ is the corresponding value in the predicted mask. The overall empirical risk ${\hat{R}}_{mask}$ is a summation of *L*_*mask*_(*y*, ŷ) on all voxels in all the 3D patches and can be efficiently minimized by using stochastic gradient descent (SGD) methods.

### 2.5. Positive-Unlabeled Learning With Box Labeled Data

When the images are only provided with bounding box annotations, it is much more difficult to learn the segmentation network *f*(**x**; θ) because the voxels inside the box can be classified as either tumor or non-tumor. A straightforward solution would be assigning all the voxels in patches coming from *S*_1_ with label 1 and labeling all the voxels in patches coming from *S*_0_ with label 0. We could then train the segmentation network using the cross-entropy loss (1), which we call the “Naive-BoxSup” method. The problem with the naive method is that some non-tumor voxels inside the box are wrongly assigned with tumor class label 1. As a result, the learned network tends to classify the voxels outside but close to the tumor boundaries as tumor voxels.

To alleviate this problem of the Naive-BoxSup, we proposed to consider segmentation from boxes as a positive-unlabeled learning problem. We can ensure that patches extracted from *S*_0_ only contains positively-labeled voxels (0 is considered the positive label), which are far away from tumor area. In the bounding box area *S*_1_, voxels in *S*_1_ can be considered as an unlabeled object. Thus, segmentation network learning from bounding box annotations is a typical positive-unlabeled learning problem, which tries to learn a classifier to model the distribution of positive data *p*_*p*_ and negative data *p*_*n*_ by using only positive labeled data and unlabeled data. In the following, we have described how we applied a recently proposed non-negative PU-Learning loss (Kiryo et al., 2017) to train our segmentation network. We chose to use this loss because the non-negative constraint on the loss makes it less prone to overfitting when a deep network is being learned.

Let *p*(*x*) denote the marginal distribution of input features corresponding to a single output *y* in the predicted segmentation mask. By stacking all the 3D patches together, we can get a sample ${\left\{\left(x_{i},y_{i}\right)\right\}}_{i=1}^{n}$. Let *p*_*p*_(*x*) = *p*(*x*|*y* = 0) and *p*_*n*_(*x*) = *p*(*x*|*y* = 1) denote the positive and negative class conditional distributions, respectively. We have

(2) $$\begin{array}{r} p\left(x\right)=\pi_{p}p_{p}\left(x\right)+\left(1-\pi_{p}\right)p_{n}\left(x\right). \end{array}$$

Equivalently, (1 − π_*p*_)*p*_*n*_(*x*) = *p*(*x*) − π_*p*_*p*_*p*_(*x*). Let *L*(*y*, ŷ) be a general loss function evaluating the distance between output and ground truth labels, which is cross-entropy loss in our case. This is denoted by

(3) $$\begin{array}{l} R_{p}^{+}\left(\theta \right)=E_{x\sim p_{p}\left(x\right)}L\left(f\left(x,\theta \right),y=0\right), \end{array}$$

(4) $$\begin{array}{l} R_{n}^{-}\left(\theta \right)=E_{x\sim p_{n}\left(x\right)}L\left(f\left(x,\theta \right),y=1\right), \end{array}$$

(5) $$\begin{array}{l} R_{p}^{-}\left(\theta \right)=E_{x\sim p_{p}\left(x\right)}L\left(f\left(x,\theta \right),y=1\right), \end{array}$$

(6) $$\begin{array}{l} R_{u}^{-}\left(\theta \right)=E_{x\sim p\left(x\right)}L\left(f\left(x,\theta \right),y=1\right). \end{array}$$

By using (), we can have an approximation of the risk on the true distribution $R\left(f\right)=E_{\left(x,y\right)\sim p\left(x,y\right)}L\left(f\left(x,\theta \right),y\right)=\pi_{p}R_{p}^{+}\left(f\right)+\pi_{n}R_{n}^{-}\left(f\right)$ by

(7) $$\begin{array}{r} R_{PU}=\pi \text{p}R_{p}^{+}\left(\theta \right)+R_{u}^{-}\left(\theta \right)-\pi \text{p}R_{p}^{-}\left(\theta \right). \end{array}$$

Theoretically, we can minimize *R*_*PU*_ to learn the optimal *theta* for our segmentation network. However, as pointed out in Kiryo et al. (2017), if the model is very flexible, empirical risks on training data will go negative, and we will suffer from serious over-fitting. Since our model is a very complicated convolutional neural network, we applied a non-negative risk estimator (Kiryo et al., 2017), as with the objective function:

(8) $$\begin{array}{r} R_{PUB}=\pi \text{p}R_{p}^{+}\left(\theta \right)+\max\left\{0,\overset{-}{\underset{u}{R}}\left(\theta \right)-\pi \text{p}R_{p}^{-}\left(\theta \right)\right\}. \end{array}$$

In practice, we need to replace the risk terms by their empirical estimates from data:

$$\begin{array}{r} \pi \text{p}\hat{R}{\text{p}}^{+}\left(\theta \right)=-\pi \text{p}\frac{1}{n}\overset{n}{\underset{i=1}{\sum }}\left(1-y_{i}\right)\cdot \log\left(1-\frac{1}{1+e^{-f\left(x_{i};\theta \right)}}\right)\\ \hat{R}{\text{u}}^{-}\left(\theta \right)=\frac{1}{n}\overset{n}{\underset{i=1}{\sum }}y_{i}\cdot \log\left(\frac{1}{1+e^{-f\left(x_{i};\theta \right)}}\right)\\ \pi \text{p}\hat{R}{\text{p}}^{-}\left(\theta \right)=-\pi \text{p}\frac{1}{n}\overset{n}{\underset{i=1}{\sum }}y_{i}\cdot \log\left(1-\frac{1}{1+e^{-f\left(x_{i};\theta \right)}}\right). \end{array}$$

The overall algorithm is shown in Algorithm 1. We used the ADAM optimizer to optimize the empirical risk. In our algorithm, we set πp = 0.75 and we set γ = 1, η = 0.5, which is a very common choice for PU learning.

**Algorithm 1:** Optimization of Our 3D-BoxSup segmentation algorithm

**Input:** training data (*x*_*i*_, *y*_*i*_);
hyperparameters 0 ≤ β ≤ πp and 0 ≤ γ ≤ 1
**Output:** model parameter θ for *f*(*x*; θ)
1: Let A be an external ADAM optimizer (Kingma and Ba, 2014)
2: **while** no stopping criterion has been met:
3: Shuffle (*x*_*i*_, *y*_*i*_) into *N* mini-batches
4: **for** *i* = 1 **to** *N*:
5: **if** $\hat{R}{\text{u}}^{-}\left(\theta \right)-\pi \text{p}\hat{R}{\text{p}}^{-}\left(\theta \right)\geq -\beta$:
6: Set gradient $\nabla_{\theta }\hat{R}\text{pu}\left(\theta \right)$
7: Update θ by A with its current step size η
8: **else**:
9: Set gradient $\nabla_{\theta }\left(\pi \text{p}\hat{R}{\text{p}}^{-}\left(\theta \right)-\hat{R}{\text{u}}^{-}\left(\theta \right)\right)$
10: Update θ by A with a discounted step size γη

## 3. Experiment

To demonstrate the effectiveness of our method, we presented a number of experiments examining different aspects of our method. After introducing the implementation details, we evaluated our methods on BraTS (Wang et al., 2017) brain tumor training dataset. We compared the segmentation performance of our 3D-BoxSup method with the Naive-BoxSup segmentation method and show advantages of our proposed method over the baseline approach.

### 3.1. Training Setting

We got all our training data from BraTS web^1^ to evaluate our method. The training data consisted of 285 patients, including segmented masks annotated by human experts. These training data were separated into two categories, including HGG and LGG, each containing 210 HGG and 75 LGG images. There is an imbalance between HGG and LGG, and the data distributions of HGG and LGG were also different, especially for TC and ET. Each patient had four sequences, which are FLAIR, T2, T1, and T1ce. In training time, we randomly split the whole training set to 80% training set and 20% as our evaluation set, and we carried out five folds testing in this manner. We only use the ground truth segmented label during evaluation. We fed all of the sequences into our network by combining them in channel dimension. Thus, our input data are in 5D, the dimensions of which are batch, sequences, width, length, and depth. The training model structure is shown in Figure 1A. To generate the training data, we followed the abovementioned section 2.2 method, and the proportion of the non-tumor voxels was π_*S*_2__ = 0.75.

**Figure 2:**
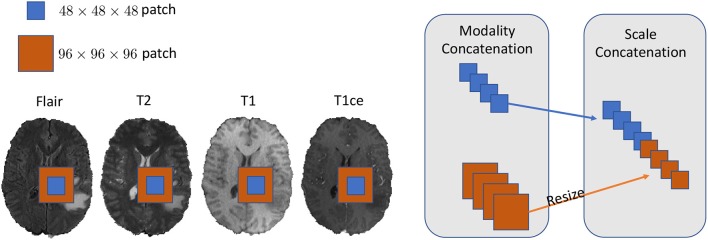
In this figure, we show the example of how to reconstruct our training data in the 2D aspect for better comprehensive as it is easy to implement in 3D level as well. We concatenated the Flair, T2, T1, and T1ce models in the channel dimension and combined extra scale information to enhance the model training.

We set our training batch size to 64, and each training patch voxel size is 48 × 48 × 48 for saving memory, which is sufficient to train the model; another aspect to the design of such a parch size is that a larger patch would contain more background voxel, which means the model would over-fit the background and would not be able learn a pattern of Whole Tumor segmentation. For the data training strategy, we randomly generated 40,000 locations as the center point of patches for each patient volume; finally, only 200 locations were selected as our training patches. To fully utilize the information from each model provided with FLAIR, T2, T1, and T1ce, we reconstructed our multi-modal data by stack theses modals in the channel dimension, which can be directly fed to convolutional neural networks (CNNs). Also, imitating the technique from (Bakas et al., 2018), we enabled the network to capture the multiscale information from data. To do so, we got the 96 × 96 × 96 patches for each modal, extracted in the same way as the 48 × 48 × 48 patches, which were two times bigger than the basic training patch; this bigger patch also belongs to the same center location as the basic training patch. Then, we resized the 96 × 96 × 96 patches to 48 × 48 × 48. Finally, we concatenated all the different models and scaled patches, which is shown in Figure 2. Thus, the input patch size of our model was *batchsize* × 8 × 48 × 48 × 48. We trained our whole network using Pytorch (Paszke et al., 2017), which is a new hybrid front-end seamlessly transitions between eager mode and graph mode to provide both flexibility and speed. NVIDIA TITAN XP GPU was applied to train our network, and the cost was about 11 gigabytes GPU RAM. The whole training process was finished in 4 h with 5 epochs, and each epoch traversed the whole training dataset. To optimize our model, we chose the common gradient descent algorithm Adam.

### 3.2. Evaluation Metrics

#### 3.2.1. Dice Coefficient

The Dice-Coefficient Score was calculated as the performance metric. This measure states the similarity between clinical Ground Truth annotations and the output segmentation of the model which are *A* and *B* respectively. Afterwards, we calculated the average of those results to obtain the overall dice coefficient of the models.

(9) $$\begin{array}{r} D=\frac{2|A⋂B|}{\left|A|+|B\right|} \end{array}$$

#### 3.2.2. Hausdorff Distance

The Hausdorff Distance is mathematically defined as the maximum distance of a set to the nearest point in the other set, in other words, how close the segmentation and the expected output are. In most evaluations, we usually adopt the 95% Hausdorff Distance, Hausdorff95, which means the chosen distance is greater or equal to exactly 95% of the other distance in two point sets.

(10) $$\begin{array}{ll} & \vec{d_{H}}\left(A,B\right)=\underset{a\in A}{\max}\underset{b\in B}{\max}d\left(a,b\right) \end{array}$$

(11) $$\begin{array}{ll} & H\left(A,B\right)=max\left\{\vec{d_{H}}\left(A,B\right),\vec{d_{H}}\left(B,A\right)\right\} \end{array}$$

### 3.3. Experimental Results

**Figure 3:**
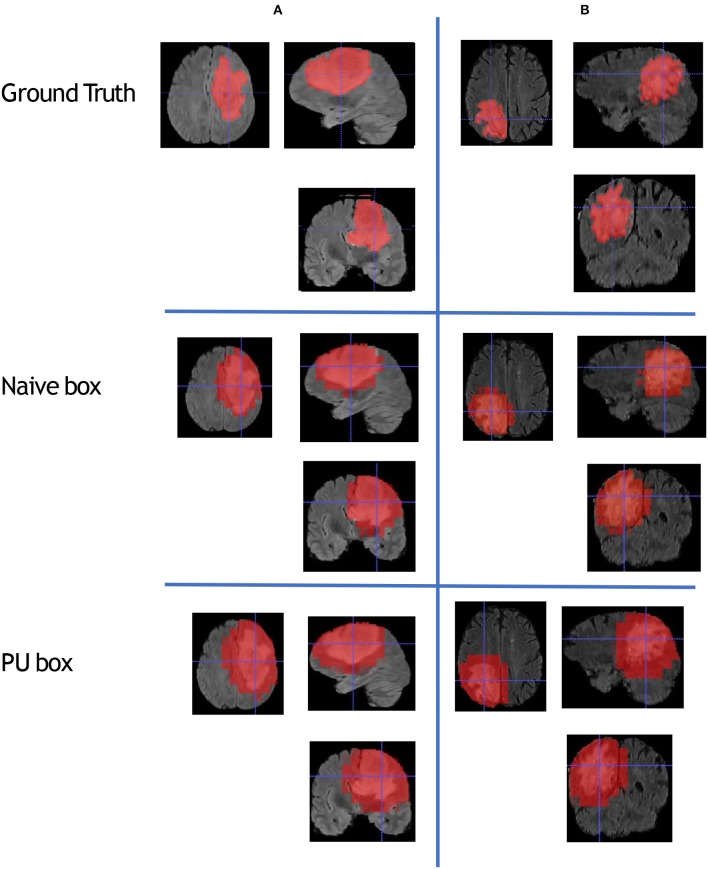
This figure shows that we randomly sampled two patients from HGG testing set and LGG testing set respectively. Each column represents the applied method, and each row is the chosen patient. The A patient is from HGG samples, and the B patient is from LGG samples. The estimated segmentation result of WT is shown by both the naive method and our proposed PU box method. To better visualize the segmented result, we provide three different views: axial, coronal, and sagittal.

To compare the model performance straightly, we gave the segmented mask generated by the baseline Naive-BoxSup method and our proposed method 3D-BoxSup, shown in Figure 3. Corresponding to the evaluated metric in section 3.2, the quantitative results are shown in Table 1. The chosen samples were randomly picked from HGG testing set and LGG testing set. As can be seen from Figure 3, our proposed 3D-BoxSup method obviously produced a more accurate segmentation mask than the Naive-BoxSup method, which produced a much more noisy mask around the tumor boundary. It seems the Naive-BoxSup over-fits the data from no-tumor area *S*_0_, which verifies that our method is able to alleviate this over-fitting and learning better from box area *S*_1_.

**Table 1:** Mean values of Dice and Hausdorff measurements of the proposed method on the BraTS 2018 validation set.

	**Dice**	**Hausdorff (mm)**	**Hausdorff95 (mm)**
	**WT**	**WT**	**WT**
Naive-BoxSup (baseline)	0.49 ± 0.04	31.213 ± 2.316	20.857 ± 1.503
3D-BoxSup (ours)	0.62 ± 0.02	28.641 ± 1.395	15.476 ± 1.132
Region Grow	0.50	39.920	29.151

*WT denotes whole tumor*.

**Figure 4:**
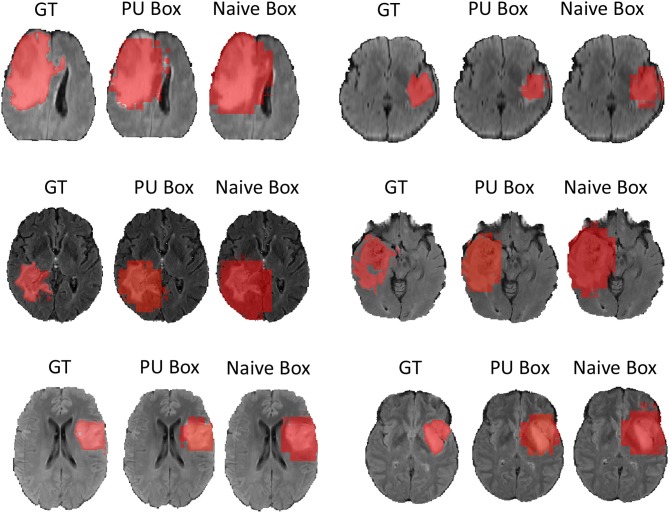
We randomly choose six patients from testing set as more example displaying and for simplicity we only show the view of axial plane.

In terms of the quantitative metrics of the Dice Score and Hausdorff Distance, our method performs better than the baseline method in each aspect, especially the Dice Score. Visually, as shown in Figure 4, out method also generates finer segmentation mask than the baseline method. The variance of our 5-folds evaluation results is also smaller than the baseline model, which means our model is more robust. Also, due to the fact that we only applied a simple post-process for fill in the hole of segmented mask, the Hausdorff Distance could be influenced by the wrong segmentation area, which is beyond the tumor area. Compared with the hand-crafted region grow method (watershed clustering Ng et al., 2006), we set the threshold of discontinuities in gray-scale to be 0.5, and the result of the region grow is shown as below. To evaluate the region grow method, we tested it on both training data and testing data as tge region grow does not need to be trained. Overall, our proposed method shows a superiority when given a weak box annotation.

## 4. Conclusion

Precisely labeled data is limited in real world, especially for medical data; it would cost a significant amount time and labor to annotate the data, and it would also require a highly qualified doctor as the annotator. Thus, we need to refer to some easily labeled data, saving time, and also explore the information derived from these weakly labeled data. In this paper, we explored one of the possibilities of weakly supervised approach on medical image segmentation. Our method is called the “3D-BoxSup,” which only acquired a 3D bounding box label for brain tumor. Compared to the traditional supervised labeled data, which needs a fine boundary for tumor annotation, our annotated data is more accessible. However, training on the box labeled data would lead to over-fitting of the background as well as a biased risk function. Box labeled data is a typical positive-unlabeled task, and we thus proposed to apply the non-negative PU risk function (Kiryo et al., 2017) to boost the performance of our model. We have shown the effectiveness of our proposed method on the data provided by BRATS challenge (Menze et al., 2015). Since our model is a general method when tackling such box labeled data, our method can be further applied to mostly if not all of the segmentation tasks.

## Data Availability Statement

The datasets analyzed in this article are not publicly available. Requests to access the datasets should be directed to BRATS challenge.

## Author Contributions

YX mainly implemented the method and conducted the experiments. MG provided the idea of this paper and contributed to the writing of the paper. JC and ZC substantially contributed to the revision. ZC performed the Region Growing experiments requested by the reviewers, and the JC helped with the writing of the revised paper. KB supervised the whole process, including the development of the concept, writing, revision, and other general advice.

## Conflict of Interest

The authors declare that the research was conducted in the absence of any commercial or financial relationships that could be construed as a potential conflict of interest.

## Footnotes

^1^https://www.med.upenn.edu/sbia/brats2018/data.html
